# Effects of tea saponin on glucan conversion and bonding behaviour of cellulolytic enzymes during enzymatic hydrolysis of corncob residue with high lignin content

**DOI:** 10.1186/1754-6834-6-161

**Published:** 2013-11-14

**Authors:** Yue Feng, Jianxin Jiang, Liwei Zhu, Linyan Yue, Junhui Zhang, Shijie Han

**Affiliations:** 1 Institute of Applied Ecology, Chinese Academy of Sciences, 72 Wenhua Road, Shenhe District, Shenyang City, Liaoning Province 110016, People's Republic of China; 2 Department of Chemistry and Chemical Engineering, Beijing Forestry University, 35 Qinghua East Road, Haidian District, Beijing 100083, People's Republic of China

## Abstract

**Background:**

Recently, interest in the utilization of corncob residue (CCR, with high lignin of 45.1%) as a feedstock for bioethanol has been growing. Surfactants have been one of the most popular additives intended to prevent the inhibitory effect of lignin on cellulolytic enzymes, thereby improving hydrolysis. In this study, the effects of biosurfactant tea saponin (TS) on the enzymatic hydrolysis of CCR and the bonding behavior of cellulolytic enzymes to the substrate were investigated. The surface tension in the supernatant was also detected to obtain information about the characteristics and stability of TS.

**Results:**

The glucose concentration was 17.15 mg/mL at 120 hours of hydrolysis with the low loading of cellulolytic enzymes (7.0 FPU/g cellulose and 10.5 BGU/g cellulose) and 5% CCR. The optimal dosage of TS was its critical micelle concentration (cmc, 1.80 mg/mL). The glucose yield was enhanced from 34.29 to 46.28 g/100 g dry matter by TS. The results indicate that TS can promote the adsorption of cellulolytic enzymes on the substrate and mediate the release of adsorbed enzymes. Meanwhile, TS improves the recovery of the cellulolytic enzymes after a hydrolysis cycle and prevents deactivation of the enzymes during the intense shaking process. The surface tension in supernatants of digested CCR with TS remained at 50.00 mN/m during the course of hydrolysis. It is interesting to note that biosurfactant TS can maintain the surface tension in supernatants, despite its digestibility by cellulolytic enzymes.

**Conclusions:**

Serving as an accelerant of lignocellulose hydrolysis, TS can also be degraded by the cellulolytic enzymes and release glucose while retaining stability, which reduces the cost of both the cellulolytic enzymes and the additive. As the glucose from the TS could be utilized by yeast, further efforts will investigate the mechanism of function and the application of TS in the production of ethanol by simultaneous saccharification and fermentation (SSF).

## Background

One of the major limitations of cellulosic ethanol production is the release of fermentable sugars from lignocellulose using cellulolytic enzymes [1,2]. Recently, interest in the utilization of corncob residue (CCR) as a feedstock for the production of bioethanol has been growing [3-6]. CCR is an industrial byproduct of furfural manufacture from corncobs, in which hemicelluloses are acid-hydrolyzed to produce furfural [7]. The cellulose and lignin present in corncobs are relatively stable during the acid hydrolyzation of hemicelluloses [8]. Therefore, the lignocellulosic residues from furfural production are mainly composed of cellulose and lignin, the components of which in the residues were on average about 43% and 45%, respectively [9-11]. It has been reported that 12 to 15 tons of CCR can be gained from the production of 1 ton of furfural, while 23 million tons of CCR have been available each year on average for alternative usage in China [12]. The advantages of using CCR in ethanol bioconversion have been reported [13]. As these residues are byproducts of hemicelluloses extraction, CCR is rich in cellulose. When acid treatment was applied during the manufacture procedure, the lignin in the CCR was less polymerized, and the cellulose was more accessible [14,15].

However, despite intensive research efforts, an efficient hydrolysis of CCR by cellulolytic enzymes is still difficult to accomplish. Lignocellulose conversion to sugar monomers on a commercial scale is hampered by the inhibitory effect of lignin [16,17]. Lignin provides a physical barrier limiting the accessibility of cellulolytic enzymes to the substrate, and the residual lignin could block the removal of the cellulase from the cellulose chain [18]. In addition, the non-productive adsorption of lignin on cellulolytic enzymes reduces the productive hydrolysis of the substrate [19]. Lignin may also directly inhibit the activities of cellulolytic enzymes [20]. Therefore, studies are focusing on additives that improve the conversion of lignocellulosic feedstock.

In recent years, surfactants have been one of the most popular additives intended to prevent the inhibitory effect of lignin on cellulolytic enzymes, thereby improving hydrolysis. A large number of reports have stated that surfactants, especially non-ionic surfactants, were the most suitable additives for improving the saccharification of lignocellulose and the recovery of cellulolytic enzymes [21-25].

However, most of the surfactants that have been studied recently were chemicals. The application of natural biosurfactants in lignocellulose hydrolysis has been less extensively investigated. Biosurfactants were more popular for their high efficiency and avirulence. It was found that biosurfactant monorhamnolipid may promote hydrolysis of NaOH-pretreated rice straw by 23.15%, and increase the stability of cellulase by 24% to 36% [26]. The improvement of the production of cellulases and xylanase from *Penicillium expansum* via the addition of biosurfactant rhamnolipid was also confirmed by Wang *et al*.. The rhamnolipid increased the activity of cellulase by 25.5% to 102.9%, and protected cellulase from degradation and inactivation. However, the reducing sugars by hydrolyzing wheat straw were not visibly increased by the rhamnolipid [27]. Zhang *et al*. also found the rhamnolipid prevented unproductive binding of enzymes to lignin [28]. The increment of 20% was found by Menon *et al*., who investigated the positive effect of sophorolipid on the hydrolysis of oat spelt xylan and wheat bran hemicelluloses with *Thermomonospora* xylanase [29].

Tea seed is an agricultural byproduct of *Camellia oleifera* Abel, which is commonly used for the production of cooking oil [30]. On average, the production of 15 million tons of tea seed oil will obtain 50 million tons of residues annually in China [31]. The defatted tea seed residues contain 11% to 17% saponin, which is usually used for detergents or organic fertilizers with low economic value [31]. Tea saponin (TS) is a type of tea seed-derived natural non-ionic biosurfactant. The TS had a weight-average molecular weight of 809.12 g/mol and contained four aglycones of L-rhamnose, D-galactose, D-glucose, and D-glucuronic acid. A critical micelle concentration (cmc) of 1.80 mg/mL and a minimum surface tension (γ_cmc_) of 43.5 mN/m were determined for the TS [31]. The cmc is the threshold value that limits the formation of micelles. Micelles will form in the solution, combining enzymes and hindering the exchange of materials, when the surfactant concentration is higher than its cmc. Nevertheless, few studies seem to have been conducted regarding the effects of biosurfactant TS on lignocellulose conversion, especially in the case of CCR saccharification.

One of the typical characteristics of a surfactant is that it can stabilize the surface tension in a solution. However, the function of surfactants in lignocellulose hydrolysis has mostly been investigated by the determination of the cellulose conversion, the enzymatic protein content, and the stability and activity of cellulolytic enzymes [27,28]. The impact of the surfactant on the surface tension in the supernatant during the course of hydrolysis has not been extensively considered.

Biosurfactant TS was investigated for its ability to improve the enzymatic hydrolysis of CCR in this study. The components of glucan and lignin in the prepared CCR were 48.3% and 45.1%, respectively. The substrate was hydrolyzed with *Trichoderma reesei* cellulase in commercial mixtures. The influence of TS on the efficiency of CCR hydrolysis and on the adsorption and recovery of cellulolytic enzymes was detected by considering the alteration of the glucose yield, protein concentrations, and enzymatic activity in the hydrolysate. To obtain the information about characteristics and stability of biosurfactant TS, the surface tension in the supernatant was also detected during the process of CCR hydrolysis.

## Results and discussion

### Effect of TS on CCR hydrolysis

**Figure 1:**
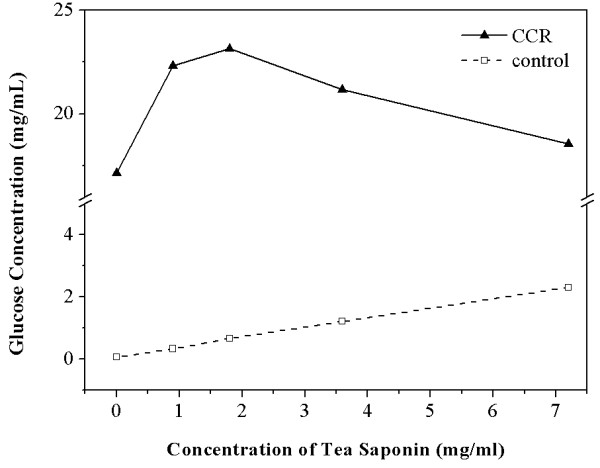
**Glucose concentration in the supernatants of digested CCR with different amounts of TS.** CCR, corncob residue; TS, tea saponin.

Biosurfactant TS was added to the mixture of CCR and cellulolytic enzymes to evaluate the effect of TS on CCR hydrolysis. Dosages of TS were compared by hydrolyzing a 5% water-insoluble solids (WIS) content containing 0 to 6 mg/mL biosurfactant. The glucose concentration was 17.15 mg/mL in the supernatant of digested CCR with cellulolytic-enzyme loading levels of 7.0 FPU/g cellulose and 10.5 BGU/g cellulose. The glucose yield was 34.29 g/100 g dry matter. The glucose concentration in the supernatant increased with the addition of TS until the TS concentration was 1.80 mg/mL, after which it decreased with the further addition of TS (Figure 1). The highest glucose concentration (23.14 mg/mL) was obtained when the TS concentration was 1.80 mg/mL. The corresponding glucose yield was 46.28 g/100 g dry matter, which is 34.97% superior to the yield in the absence of TS. The results indicate that the addition of biosurfactant TS in a concentration lower than 1.80 mg/mL can enhance CCR hydrolysis. With a concentration higher than 1.80 mg/mL, hydrolysis would be interrupted. The same result was reported by Zhang *et al*., who used PEG4000 as surfactant to improve hydrolysis of the acid steam-exploded corn straw, the pure microcrystalline cellulose, and the bagasse sulfite pulp [32]. The optimal concentration of TS in the supernatants of digested CCR was 1.80 mg/mL, the value of which is the cmc of the biosurfactant TS. The CCR saccharification was not optimally enhanced when the TS concentration was lower than its cmc. Micelles, which are obstacles to intermolecular transfer, will form if the concentration of a surfactant has passed its cmc. The enzymatic hydrolysis of CCR will be hindered by TS when the TS concentration is greater than 1.80 mg/mL. Therefore, the cmc is the optimal dosage of TS in CCR hydrolysis.

It is interesting to note that glucose could be released in the control group without the addition of CCR (Figure 1). Glucose production in the solution of TS with cellulolytic-enzyme loading levels of 7.0 FPU/g cellulose and 10.5 BGU/g cellulose increased when more TS was added into the mixture. The glucose concentration reached 2.31 mg/mL when the dosage of TS was 7.20 mg/mL. This result indicates that cellulolytic enzymes can release glucose from the biosurfactant TS. However, the glucose released from the TS was eliminated from the calculation of the substrate saccharification yield in the above CCR enzymatic hydrolysis process involving TS to ensure the applicability and accuracy of the experimental data.

**Figure 2:**
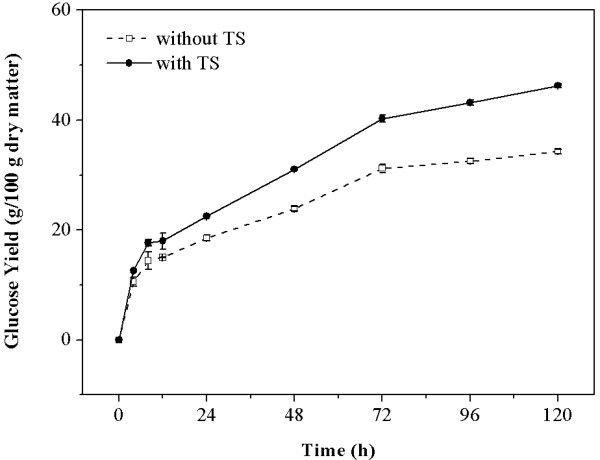
**Glucose yield in the supernatants of digested CCR with and without TS.** CCR, corncob residue; TS, tea saponin.

The glucose yields over time in the supernatants of digested CCR were measured to evaluate the effect of 1.80 mg/mL of TS on glucose production. As shown in Figure 2, the increment of glucose yields is obvious in both supernatants with and without TS throughout the hydrolysis process. With the addition of TS, the glucose released from the CCR increased from 15.01 to 18.02 g/100 g dry matter at 12 hours. The increment of the glucose yield per hour was 0.25 g/100 g dry matter during the time period of 0 to 12 hours. At 72 hours, the glucose yield increased from 31.20 to 40.27 g/100 g dry matter with the addition of TS. The increment of the glucose yield per hour was 0.10 g/100 g dry matter during the time period of 12 to 72 hours. At 120 hours of hydrolysis, the glucose yield increased from 34.29 to 46.28 g/100 g dry matter with the addition of TS. The increment of the glucose yield per hour was 0.06 g/100 g dry matter during the time period of 72 to 120 hours. These results suggest that TS can enhance CCR hydrolysis throughout all stages of reaction. The most significant enhancement was observed in the initial 12 hours, which implied that the feedback inhibition of glucose on the CCR hydrolysis was remarkable in spite of the participation of biosurfactant TS.

### Effect of TS on protein concentrations in the supernatants of digested CCR

**Figure 3:**
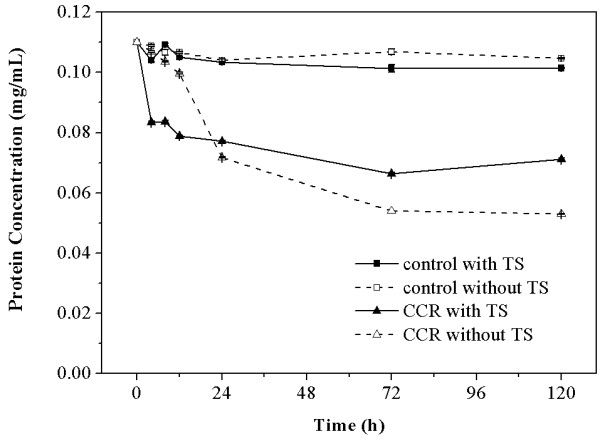
**Protein concentration in the supernatants of digested CCR with and without TS.** CCR, corncob residue; TS, tea saponin.

As TS could enhance the conversion of CCR, the protein concentrations in the supernatants of digested CCR and control with and without the addition of TS (1.80 mg/mL) were measured to further investigate the beneficial function of TS in the CCR hydrolysis process. The protein concentrations in the supernatants during the time period of 0 to 120 hours are shown in Figure 3 to illustrate the alteration of the protein concentrations in the supernatants throughout the hydrolysis process. The initial protein concentration in the supernatant was detected to be 0.11 ± 0.005 mg/mL. The protein concentrations rapidly decreased by 30% to 35% during the initial 24 hours in the supernatants of digested CCR with and without TS, which indicates that adsorption of the cellulolytic enzymes on the substrate mainly occurs at the front stage of hydrolysis. There was a significant difference in the alteration of protein concentrations with and without TS. With the addition of TS, the protein concentration in the supernatants of digested CCR rapidly decreased to 0.08 ± 0.004 mg/mL at 4 hours. It experienced a small upward fluctuation at 8 hours, and then it gradually decreased to its lowest value of 0.07 ± 0.003 mg/mL at 72 hours. The protein concentration increased by 4.31% during the later phase of hydrolysis (72 to 120 hours), which suggests that the adsorbed cellulolytic enzymes could be released into the supernatant at the end of hydrolysis. For comparison, the protein concentration in the supernatants of digested CCR without TS was 0.11 ± 0.003 mg/mL at 4 hours, which suggests that the adsorption of enzymes on the substrate is tardy in the absence of TS. It decreased to 0.07 ± 0.001 mg/mL at 24 hours, then it kept reducing until reaching a value of 0.05 ± 0.000 mg/mL at 120 hours, which suggests that the adsorbed cellulolytic enzymes could not desorb into the supernatant at the end of saccharification. The results indicate that TS can promote the efficiency of adsorption of cellulolytic enzymes on the CCR at the beginning of hydrolysis and lead to the release of adsorbed enzymes at the end of hydrolysis. The similar function of the chemical-surfactant Tween 80 was reported by Okino *et al*. [33]. Moreover, the free protein concentrations in the supernatants of control with and without TS were similar. This result indicates that adsorption of cellulolytic enzymes on the biosurfactant TS was not obvious, although TS can be degraded by the cellulolytic enzymes during the process of hydrolysis.

### Effect of TS on filter paper activity in the supernatants of digested CCR

**Figure 4:**
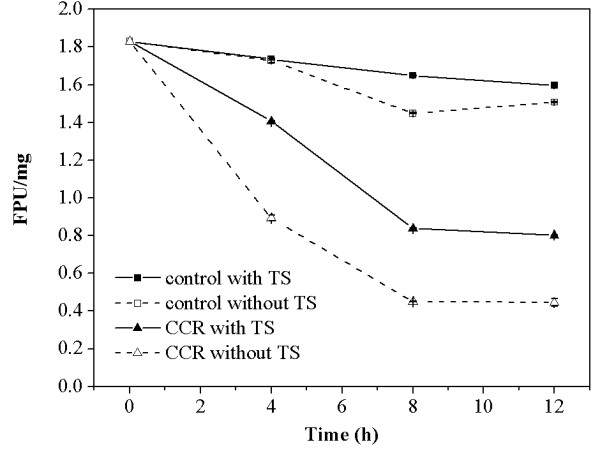
**FPA in the supernatants of digested CCR with and without TS.** CCR, corncob residue; FPA, filter paper activity; TS, tea saponin.

It was found that biosurfactant TS could promote the desorption of cellulolytic enzymes from the lignocellulosic substrate. It has been reported that the non-productive adsorption between cellulolytic enzymes and lignin, a constituent part of lignocelluloses, is one explanation for the decrease of glucose conversion [19]. Therefore, the filter paper activity (FPA) of free cellulolytic enzymes in the supernatants of digested CCR and control with and without the addition of TS (1.80 mg/mL) were measured to investigate another function of TS in the CCR hydrolysis process. The FPA during the time period of 0 to 12 hours is shown in Figure 4 to illustrate the alteration of FPA in the supernatants during the hydrolysis process. As Figure 4 shows, the initial FPA in the supernatant was detected to be 1.83 ± 0.021 FPU/mg. FPA of free cellulolytic enzymes decreased to 0.89 ± 0.016 FPU/mg at 4 hours in the supernatants of digested CCR, which implies that the released enzyme will be inactivated after its interaction with CCR. There was a significant difference in the loss of FPA of the free enzymes with and without TS in the supernatants of digested CCR. With the addition of TS, the FPA decreased to 1.41 ± 0.001 FPU/mg at 4 hours in the supernatants of digested CCR. The recovery of cellulase is improved by 28.10% with TS. The FPA of 0.80 ± 0.002 FPU/mg was observed at 12 hours in the supernatant with added TS. The corresponding FPA of the cellulase was 79.89% higher than FPA in the supernatant without TS at the same time. The results demonstrate that TS can improve the recovery of cellulase activity in the supernatants of digested CCR, which resembles the result determined by Yang *et al*. [34]. Moreover, the FPA also remained stable in the control supernatant with the addition of TS after 4 hours. The FPA in control digest at 12 hours was 1.60 ± 0.014 FPU/mg with the addition of TS, while it decreased to 1.51 ± 0.003 FPU/mg without TS. The result indicates the effect of TS on the stability of the cellulolytic enzymes during the intense shaking process.

### Effect of TS on surface tension in the supernatants of digested CCR

**Figure 5:**
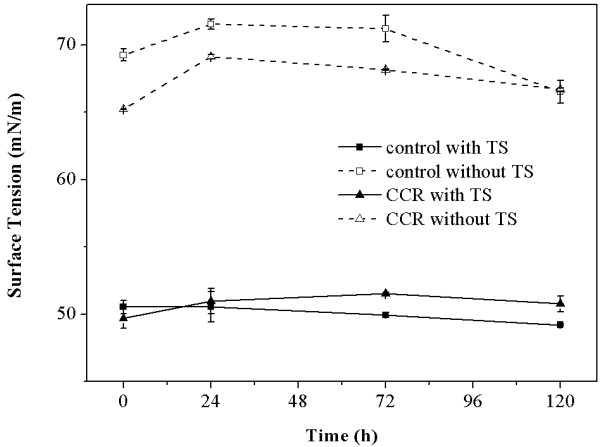
**Surface tension in the supernatants of digested CCR with and without TS.** CCR, corncob residue; TS, tea saponin.

Another significant effect of a surfactant, the maintenance of stable surface tension, is well known. The previous study shows that TS can be degraded by cellulolytic enzymes. Therefore, the surface tension in the supernatants of digested CCR and control with TS was assayed to test the stability of this non-ionic biosurfactant. As shown in Figure 5, the surface tension in both the CCR and control supernatants without TS clearly fluctuated, the numbers of which are around 65.22 to 71.54 mN/m during the hydrolysis process. With the addition of TS, the surface tension in both the CCR and control supernatants was reduced and remained at approximately 50.00 mN/m during the course of hydrolysis. It is interesting to note that the biosurfactant TS could maintain the surface tension in both the CCR and control supernatants, despite its hydrolyzation by cellulolytic enzymes. The results revealed that TS has an excellent stability with respect to cellulase and a beneficial effect on the stabilization of the surface tension during the hydrolysis process.

### Process of CCR hydrolysis with the participation of TS

**Figure 6:**
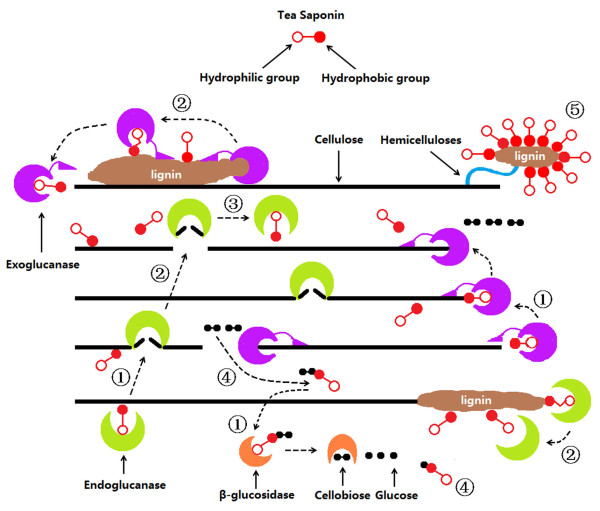
**Schematic diagram representing the process of CCR hydrolysis with the participation of TS. (1)** Enzymes and substrates are attracted together by the TS; **(2)** TS promotes the release of enzymes non-productively binding on the substrate; **(3)** TS led to a higher recovery of enzymatic FPAs at the end of hydrolysis; **(4)** TS homogenize organic matter in solution with its hydrophilic and hydrophobic groups; and **(5)** the hydrophobic interaction between TS and lignin. CCR, corncob residue; FPA, filter paper activity; TS, tea saponin.

A surfactant is composed of a hydrophilic group and a hydrophobic group, and it acts as an accelerant for lignocelluloses hydrolysis in a solid-liquid phase system [35]. As shown in Figure 6, the hydrophilic group can combine the cellulolytic enzymes in the liquid supernatant, while the hydrophobic group can bond the solid substrates. Enzymes and substrates might be attracted together by the surfactant, which enhances the adsorption of enzymes and the accessibility of substrates. TS also appear to promote the release of enzymes binding on the substrate by its hydrophobic interaction with lignin. The surfactant may also display a competitive adsorption with the cellulolytic enzymes, thus reducing the binding of the enzymes on the lignin [28]. TS enhance the adsorption and desorption between the enzymes and the substrate, which is one reason for the improved hydrolysis of CCR. The addition of TS also led to a higher recovery of FPA at the end of hydrolysis. The above results suggest that TS can be used to promote desorption and protect enzymatic activity for recycling after a hydrolysis cycle. TS may homogenize organic matter in solution with its hydrophilic and hydrophobic groups, leading to the dispersal of sugars and the alleviation of feedback inhibition to the cellulolytic enzyme. It can also decrease the surface tension in supernatants, which reduces the energy consumption of hydrolysis and protects the cellulolytic enzymes from deactivation on the surface of the liquid phase.

Therefore, the positive effect of TS on the bonding and recovery of cellulolytic enzymes, accessibility of lignocelluloses, and homogenization of hydrolysates may provide the explanation for the improved conversion of CCR in the presence of TS. However, more experimental studies are needed to confirm the comments.

## Conclusions

Biosurfactant TS can promote CCR saccharification by 34.97%. The functions of TS in CCR hydrolysis were investigated through the detection of the protein concentrations, FPAs, and surface tensions in supernatants. The results indicate that TS can improve the combination of the enzymes and the substrates, and the recovery and stability of the enzymes for recycling, thereby enhancing the hydrolysis of the lignocellulose substrate. Serving as an accelerant of lignocellulose hydrolysis, TS can also be degraded by the cellulolytic enzymes and release glucose while retaining stability, which reduces the cost of both cellulases and additives. As the glucose from the TS could be utilized by yeast, further efforts will investigate the mechanism of TS action and application of TS in the production of ethanol by simultaneous saccharification and fermentation (SSF).

## Materials and methods

### Substrate preparation

The CCR was kindly supplied by the Chunlei Furfural Corporation (Hebei, China). The residues, which had a pH of 2 to 3 initially, were immersed in a 1% NaOH solution for half an hour and then washed with fresh tap water until neutral. The samples were dried at 50°C for 12 hours and milled to a size of below 40 to 60 mesh. According to the National Renewable Energy Laboratory (NREL) methods that were employed to determine and calculate the constituent contents of the samples, the proportions of glucan, xylan, and lignin in the CCR were 48.3%, 3.6%, and 45.1%, respectively [12]. Whatman No 1 filter paper was purchased from the Sigma-Aldrich (Beijing, China).

### Cellulolytic enzymes

Celluclast 1.5 L, a cellulase preparation from *Trichoderma reesei*, and Novozyme 188, a β-glucosidase preparation from *Aspergillus niger*, were purchased from Novozymes investment Co, Ltd (Beijing, China). The activity of Celluclast 1.5 L was detected to be 74 FPU/mL. The activity of Novozyme 188 was detected to be 175 BGU/mL.

### Biosurfactant preparation

Biosurfactant TS was isolated and purified from the defatted seed of *Camellia oleifera* Abel in the laboratory [31]. The TS had a weight-average molecular weight of 809.12 g/mol and contained four aglycones of L-rhamnose, D-galactose, D-glucose, and D-glucuronic acid. A cmc of 1.80 mg/mL and γ_cmc_ of 43.5 mN/m were determined for the TS [31].

### Hydrolysis of CCR with cellulolytic enzymes

Enzymatic hydrolysis was accomplished with an enzyme loading of 7.0 FPU/g cellulose to evaluate the hydrolytic potential of the CCR by the commercial cellulase preparations with the addition of biosurfactant TS. The efficiency of the hydrolysis was improved by β-glucosidase supplementation with Novozyme 188 at an enzyme loading of 10.5 BGU/g cellulose. The cellulosic substrates were diluted with the addition of 0.1 mol/L sodium acetate buffer (pH 4.8) to 50 g substrate/L in a total reaction volume of 100 mL. Saccharification was performed at 45°C on a rotary shaker at 180 rpm for 120 hours. The samples were withdrawn and centrifuged at 10,000 × *g* for 10 minutes. The supernatants were withdrawn for the evaluations of FPAs, protein concentrations, and surface tension. The supernatants were also filtered through 0.2 μm filters and diluted as indicated for neutral sugar analysis. Control hydrolysis without any substrate was performed to avoid the release of sugars from the TS and cellulolytic enzymes.

### Analytical methods

The FPA was evaluated using the standard method of the International Union of Pure and Applied Chemistry (IUPAC) [36]. The β-glucosidase activity (BGA) was determined using the modified Berghem’s method [37]. The enzymatic protein concentration was determined by the Bradford method using BSA as a standard [38]. The above experiments were repeated three times, and the data presented are the mean values.

The samples were filtered through a 0.22 μm filter and diluted appropriately by distilled water. Glucose and cellobiose were analyzed by HPLC (Waters 2695e, Waters, Milford, MA, USA) with an Aminex HPX-87P column (300 × 7.8 mm, Bio-Rad, Hercules, CA, USA) at 85°C and by a refractive index detector at 35°C. The injection volume of the sample was 10 μL, and distilled water was used as the eluent at a flow rate of 0.6 mL/min. The glucose yield was expressed as the weight of the glucose released in the supernatant to the 100 g dry matter of the loading substrate. The sugar determination was performed in duplicate under the same conditions, and the average values were computed. The standard deviations were less than 3.6%.

The surface tensions of the supernatant samples were determined according to Jian’s method on the automatic tension meter (model JK99B, Zhongchen digital technology equipment Co, Ltd, Shanghai, China) at 20°C in an aqueous medium [31].

## Abbreviations

BGA: β-glucosidase activity; BGU: β-glucosidase unit; BSA: Bovine serum albumin; CCR: Corncob residue; cmc: Critical micelle concentration; FPA: Filter paper activity; FPU: Filter paper unit; HPLC: High performance liquid chromatography; IUPAC: International union of pure and applied chemistry; NREL: National renewable energy laboratory; PEG: Polyethylene glycol; SSF: Simultaneous saccharification and fermentation; TS: Tea saponin; WIS: Water-insoluble solids; γcmc: Minimum surface tension.

## Competing interests

The authors declare that they have no competing interests.

## Authors’ contributions

YF designed experiments, carried out the analysis of glucose yield and FPA, and wrote the manuscript. JJ conceived the study. LZ participated in the measurement of surface tension. LY carried out the analysis of protein concentration. JZ helped with statistical design and analysis. SH participated in the coordination and edited the manuscript. All authors read and approved the final manuscript.

## References

[B1] Weiss N Börjesson J Pedersen LS Meyer AS Enzymatic lignocellulose hydrolysis: Improved cellulase productivity by insoluble solids recycling Biotechnol Biofuels 2013 6 5 10.1186/1754-6834-6-5 23336604 PMC3560254

[B2] Lu J Weerasiri RR Lee I Carbon nanotubes tuned foam structures as novel nanostructured biocarriers for lignocellulose hydrolysis Biotechnol Lett 2013 35 181 188 10.1007/s10529-012-1066-5 23070625

[B3] Tang Y Bu L He J Jiang J L(+)-Lactic acid production from furfural residues and corn kernels with treated yeast as nutrients Eur Food Res Technol 2013 236 365 371 10.1007/s00217-012-1865-x

[B4] Liu HQ Feng Y Zhao DQ Jiang JX Evaluation of cellulases produced from four fungi cultured on furfural residues and microcrystalline cellulose Biodegradation 2012 23 465 472 10.1007/s10532-011-9525-6 22116409

[B5] Xing Y Bu LX Wang K Jiang JX Pretreatment of furfural residues with alkaline peroxide to improve cellulose hydrolysis and characterization of isolated lignin Cellulose Chem Technol 2012 46 249 260

[B6] Liu HQ Feng Y Zhao DQ Jiang JX Influence of cellulose content on the enzyme activity in the saccharification digests of furfural residues Asia-Pac J Chem Eng 2012 7 s275 s279

[B7] Mao L Zhang L Gao N Li A FeCl_3_ and acetic acid co-catalyzed hydrolysis of corncob for improving furfural production and lignin removal from residue Bioresource Technol 2012 123 324 331 10.1016/j.biortech.2012.07.058 22940337

[B8] Yu H Tang Y Xing Y Zhu L Jiang J Improvement of the enzymatic hydrolysis of furfural residues by pretreatment with combined green liquor and hydrogen peroxide Bioresource Technol 2013 147 29 36 10.1016/j.biortech.2013.08.013 23985372

[B9] Bu L Xing Y Yu H Gao Y Jiang JX Comparative study of sulfite pretreatments for robust enzymatic saccharification of corn cob residue Biotechnol Biofuels 2012 5 87 10.1186/1754-6834-5-87 23206858 PMC3537520

[B10] Cheng K Wang W Zhang J Zhao Q Li J Xue J Statistical optimization of sulfite pretreatment of corncob residues for high concentration ethanol production Bioresource Technol 2011 102 3014 3019 10.1016/j.biortech.2010.09.117 21067916

[B11] Liu K Lin X Yue J Li X Fang X Zhu M Lin J Qu Y Xiao L High concentration ethanol production from corncob residues by fed-batch strategy Bioresource Technol 2010 101 4952 4958 10.1016/j.biortech.2009.11.013 20004568

[B12] Bu L Tang Y Gao Y Jian H Jiang JX Comparative characterization of milled wood lignin from furfural residues and corncob Chem Eng J 2011 175 176 184

[B13] Xing Y Bu LX Zhu LW Jiang JX Ultrasound-assisted enzymatic hydrolysis of furfural residues after alkaline peroxide pretreatment Chem Ind Forest Prod 2012 32 47 52

[B14] Zhang L Li T Wang L Li SZ Enzymatic hydrolysis of corncob residues of furfural manufacture and optimum conditions for cellulose conversion Trans Chin Soc Agric Eng 2009 10 226 230

[B15] Sun R Song X Sun R Jiang J Effect of lignin content on enzymatic hydrolysis of furfural residues Bioresources 2011 6 317 328

[B16] Nakagame S Chandra RP Kadla JF The characterization and possible role of lignin from steam and organosolv pretreated substrates on enzymatic hydrolysis [abstract] 32nd Symposium on Biotechnology for Fuels and Chemicals: 19–22, April, 2010 2010 Florida 8 20

[B17] Lee SH Doherty TV Linhardt RJ Dordick JS Ionic liquid-mediated selective extraction of lignin from wood leading to enhanced enzymatic cellulose hydrolysis Biotechnol Bioeng 2009 102 1368 1376 10.1002/bit.22179 19090482

[B18] Alvira P Tomás-Pejó E Ballesteros M Negro MJ Pretreatment technologies for an efficient bioethanol production process based on enzymatic hydrolysis: A review Bioresource Technol 2010 101 4851 4861 10.1016/j.biortech.2009.11.093 20042329

[B19] Kumar P Barrett DM Delwiche MJ Stroeve P Methods for pretreatment of lignocellulosic biomass for efficient hydrolysis and biofuel production Ind Eng Chem Res 2009 48 3713 3729 10.1021/ie801542g

[B20] Dyk JSV Pletschke BI A review of lignocellulose bioconversion using enzymatic hydrolysis and synergistic cooperation between enzymes-factors affecting enzymes, conversion and synergy Biotechnol Adv 2012 30 1458 1480 10.1016/j.biotechadv.2012.03.002 22445788

[B21] Zhou H Lou H Yang D Zhu JY Qiu X Lignosulfonate to enhance enzymatic saccharification of lignocelluloses: role of molecular weight and substrate lignin Ind Eng Chem Res 2013 52 8464 8470 10.1021/ie401085k

[B22] Cao S Aita GM Enzymatic hydrolysis and ethanol yields of combined surfactant and dilute ammonia treated sugarcane bagasse Bioresource Technol 2013 131 357 364 10.1016/j.biortech.2012.12.170 23376200

[B23] Eckard AD Muthukumarappan K Gibbons W Enzyme recycling in a simultaneous and separate saccharification and fermentation of corn stover: A comparison between the effect of polymeric micelles of surfactants and polypeptides Bioresource Technol 2013 132 202 209 10.1016/j.biortech.2013.01.018 23411449

[B24] Eriksson T Borjesson J Tjerneld F Mechanism of surfactant effect in enzymatic hydrolysis of lignocellulose Enzyme Microb Technol 2002 31 353 364 10.1016/S0141-0229(02)00134-5

[B25] Jeya M Kalyani D Dhiman SS Kim H Woo S Kim D Lee J Saccharification of woody biomass using glycoside hydrolases from *Stereum hirsutum* Bioresource Technol 2012 117 310 316 10.1016/j.biortech.2012.03.047 22617039

[B26] Yuan X Liang Y Zeng G Wang W Hydrolysis of pretreated rice straw with surfactants at low cellulase dosage[http://www.paper.edu.cn/releasepaper/content/201001-1052]

[B27] Wang H Fan B Li C Liu S Li M Effects of rhamnolipid on the cellulase and xylanase in hydrolysis of wheat straw Bioresource Technol 2011 102 6515 6521 10.1016/j.biortech.2011.02.102 21478013

[B28] Zhang Q He G Wang J Cai W Xu Y Mechanisms of the stimulatory effects of rhamnolipid biosurfactant on rice straw hydrolysis Appl Energy 2009 86 s233 s237

[B29] Menon V Prakash G Prabhune A Rao M Biocatalytic approach for the utilization of hemicellulose for ethanol production from agricultural residue using thermostable xylanase and thermotolerant yeast Bioresource Technol 2010 101 5366 5373 10.1016/j.biortech.2010.01.150 20227273

[B30] Zhu XY Lin HM Chen X Xie J Wang P Mechanochemical-assisted extraction and antioxidant activities of kaempferol glycosides from *Camellia oleifera* Abel. meal J Agric Food Chem 2011 59 3986 3993 10.1021/jf1042689 21410254

[B31] Jian H Liao X Zhu L Zhang W Jiang J Synergism and foaming properties in binary mixtures of a biosurfactant derived from *Camellia oleifera* Abel and synthetic surfactants J Colloid Interface Sci 2011 359 487 492 10.1016/j.jcis.2011.04.038 21543081

[B32] Zhang M Ouyang J Liu B Yu H Jiang T Cai C Li X Comparison of hydrolysis efficiency and enzyme adsorption of three different cellulosic materials in the presence of poly (ethylene glycol) Bioenerg Resdoi:10.1007/s12155-013-9334-3

[B33] Okino S Ikeo M Ueno Y Taneda D Effects of Tween 80 on cellulase stability under agitated conditions Bioresource Technol 2013 142 535 539 10.1016/j.biortech.2013.05.078 23765004

[B34] Yang M Zhang A Liu B Li W Xing J Improvement of cellulose conversion caused by the protection of Tween-80 on the adsorbed cellulose Biochem Eng J 2011 56 125 129 10.1016/j.bej.2011.04.009

[B35] Bai Y Lin D Wu F Wang Z Xing B Adsorption of Triton X-series surfactants and its role in stabilizing multi-walled carbon nanotube suspensions Chemosphere 2010 79 362 367 10.1016/j.chemosphere.2010.02.023 20206374

[B36] Ghose TK Measurement of cellulaseactivities Pure Appl Chem 1986 59 257 268

[B37] Kovacs K Szakacs G Zacchi G Comparative enzymatic hydrolysis of pretreated spruce by supernatants, whole fermentation broths and washed mycelia of *Trichoderma reesei* and *Trichoderma atroviride* Bioresource Technol 2009 100 1350 1357 10.1016/j.biortech.2008.08.006 18793835

[B38] Bradford MM A rapid and sensitive method for the quantitation of microgram quantities of protein utilizing the principle of protein-dye binding Anal Biochem 1976 72 248 254 10.1016/0003-2697(76)90527-3 942051

